# Strong concordance between percent inhibition in oocyst and sporozoite intensities in a *Plasmodium falciparum* standard membrane-feeding assay

**DOI:** 10.1186/s13071-019-3470-3

**Published:** 2019-05-06

**Authors:** Kazutoyo Miura, Bruce J. Swihart, Bingbing Deng, Luwen Zhou, Thao P. Pham, Ababacar Diouf, Michael P. Fay, Carole A. Long

**Affiliations:** 1 Laboratory of Malaria and Vector Research, National Institute of Allergy and Infectious Diseases, National Institutes of Health, 12735 Twinbrook Parkway, Rockville, MD 20852 USA; 2 Biostatistics Research Branch, National Institute of Allergy and Infectious Diseases, National Institutes of Health, 5601 Fishers Lane, Rockville, MD 20852 USA

**Keywords:** Malaria, Transmission-blocking vaccine, Standard membrane-feeding assay, Oocyst, Sporozoite

## Abstract

**Background:**

Effective malaria transmission-blocking vaccines (TBVs) can support malaria eradication programmes, and the standard membrane-feeding assay (SMFA) has been used as a “gold standard” assay for TBV development. However, in SMFA, the inhibitory activity is commonly measured at oocyst stage of parasites, while it is the sporozoites which transmit malaria from a mosquito to a human. A handful of studies have shown that there is a positive correlation between oocyst and sporozoite intensities. However, no study has been completed to compare inhibition levels in oocyst and sporozoite intensities in the presence of transmission-blocking (TB) antibodies.

**Results:**

*Plasmodium falciparum* NF54 gametocytes were fed to *Anopheles stephensi* mosquitoes with or without anti-Pfs25 or anti-Pfs48/45 TB antibodies in 15 independent assays. For each group, a portion of the mosquitoes was dissected for oocyst counts (day 8 after feed), and a portion of the remaining mosquitoes was dissected for sporozoite counts (day 16). This study covered a large range of oocyst and sporozoite intensities: 0.2 to 80.5 on average for oocysts, and 141 to 77,417 for sporozoites. The sporozoite data were well explained by a zero-inflated negative binomial model, regardless of the presence or absence of TB antibodies. Inhibition levels in both oocyst and sporozoite intensities were determined within the same groups in 9 independent assays. When the level of inhibition in sporozoite number (expressed as Log Mean Ratio, LMR; average number in a control group was divided by the one in a test group, then took a log of the ratio) was plotted against LMR in oocyst number, the best-fit slope of a linear regression was not different from 1 (the best estimate, 1.08; 95% confidence interval, 0.87 to 1.29). Furthermore, a Bland–Altman analysis showed a strong agreement between inhibitions in oocysts and in sporozoites.

**Conclusions:**

The results indicate that percent inhibition in oocyst intensity of a test sample can be directly converted to % inhibition in sporozoite intensity in *P. falciparum* SMFA. Therefore, if sporozoite intensity determines transmission rate from mosquitoes to humans, the percent inhibition in oocyst intensity measured by SMFA can be used to estimate the TBV efficacy.

**Electronic supplementary material:**

The online version of this article (10.1186/s13071-019-3470-3) contains supplementary material, which is available to authorized users.

## Background

Due to the expanded application of anti-malarial control measures, such as insecticide-treated nets, rapid diagnosis, and antimalarial drugs, the mortality of malaria has been reduced significantly in the last 15–20 years. Despite this great progress, there were still 445,000 estimated malaria related deaths in 2016, mostly due to *Plasmodium falciparum* [1]. As resistance against existing drugs and insecticides has been observed in many endemic areas [1], multiple novel tools are likely to be required to achieve the ultimate goal of malaria eradication. Transmission-blocking vaccines (TBVs) are designed to induce antibodies in human hosts against sexual stage malaria antigens or to antigens found in the mosquito vector [2]. When gametocyte-stage parasites from human hosts are taken up by a mosquito, the parasites egress from erythrocytes, differentiate into male or female gametes, fertilize, and form zygotes. The zygotes further differentiate to ookinetes, which penetrate the midgut epithelium of mosquitoes, then become oocysts. Each oocyst can produce many sporozoites, and eventually some of the sporozoites which move to salivary glands are injected into the next human hosts.

There are several biological assays to determine the functionality of TBV-induced antibodies [3], and the standard membrane-feeding assay (SMFA) is considered one of the “gold standard” assays. In this assay, a mixture of cultured *P. falciparum* gametocytes and test antibodies are fed to *Anopheles* mosquitoes through a membrane-feeding apparatus, and the mosquitoes are dissected approximately one week later to enumerate oocysts in the midgut. While the precise mechanism of action for the TBV-induced antibodies has not been fully elucidated, the antibodies, which are ingested with gametocyte-stage parasites, should inhibit the parasite development before oocyst formation, as antibody remains active only for 24–36 hours in the mosquitoes [4]. Therefore, it is logical to assess the efficacy of TBV-induced antibodies at the oocyst stage. However, to estimate vaccine efficacy (prevention of the infection from mosquitoes to humans), it may be hypothesized that percent inhibition in intensity (% transmission reducing activity, %TRA) at the sporozoite stage could be a better predictor than %TRA in oocyst number, unless a vaccine can induce 100% blocking antibodies (i.e. 100% TRA at the oocyst stage). Even when a strong vaccine is developed, vaccine-induced antibody titers are likely to wane with time. Thus, it is crucial to evaluate %TRA in oocysts and that in sporozoites under an “imperfect (i.e. < 100% TRA)” condition.

As expected, a positive correlation between oocyst and sporozoite intensities has been observed in multiple studies with *P. falciparum* [5–7], *P. vivax* [7–9] and *P. berghei* [10]. However, all of those studies were conducted in the absence of transmission-blocking (TB) antibodies. In this study, using anti-Pfs25 or anti-Pfs48/45 TB antibodies, %TRA in oocysts was compared with %TRA in sporozoites in the same group. The results indicate that %TRA in oocysts can be directly converted to %TRA in sporozoites in *P. falciparum* SMFA.

## Methods

### SMFA

The standardized methodology for performing the SMFA has been described previously [11]. Unsynchronized *P. falciparum* NF54 parasites were maintained with daily medium change, but without addition of fresh red blood cells (RBCs), for16–18 days to induce mature gametocytes. On the day of feed, the parasite cultures were diluted with fresh RBCs and fresh culture medium to adjust to 50% haematocrit and 0.15–0.20% stage V gametocytaemia. The 200 μl of diluted parasite cultures were mixed with 60 μl of a test or control sample, and the final mixture was immediately fed to ~50 female *Anopheles stephensi* (Nijmegen strain, three to six days-old) mosquitoes through a membrane-feeding apparatus for 20–30 minutes. The *An. stephensi* mosquitoes were provided from the Catholic University of the Netherlands in 1985 and have been cultured at NIH for > 30 years. The insectary was set at 27 °C, 75% humidity, and 12 h of light and 12 h of darkness (with 15 minutes of dusk or dawn during the transition). A portion of the mosquitoes was dissected on day 8 [*n* = 20 per “Container of Mosquitoes” (COM)], and the midguts were stained with 0.05% mercurochrome to enumerate the oocysts in the individual midguts. Throughout the paper, COM refers to a group of mosquitoes which were housed in the same container and were fed the same final mixture of gametocyte cultures and control/test antibodies. Salivary glands from each mosquito were collected on day 16 into a 1.5 ml tube with 50 µl of 1× phosphate-buffered saline (1× PBS), pH 7.4. After pipetting up and down to release sporozoites from the salivary glands in the tube, the sporozoite mixture was transferred to a hemacytometer for counting. The minimum detection limit was 250 sporozoites per mosquito. Only midguts or salivary glands from mosquitoes with any eggs at the time of dissection were analyzed. Mouse anti-Pfs25 monoclonal antibody (mAb, 4B7) [12], mouse anti-Pfs48/45 mAb (3E12) [13], and mouse anti-Pfs25 polyclonal antibody [14] were utilized as TB antibodies. As a control, a group of mosquitoes were fed with normal mouse IgG or normal human serum (NHS), and inhibition level of TB antibodies group(s) was calculated against the normal mouse IgG or NHS control COM in the same feed. All oocyst and sporozoite counts in individual mosquitoes are shown in Additional file 1: Table S1. In one feeding experiment (Feed # 104_1), salivary glands from multiple mosquitoes were pooled for each COM, and the sporozoite intensity of the group was determined. The human serum and red blood cells used for the gametocyte cultures and feeding experiments were purchased from Interstate Blood Bank (Memphis, TN, USA).

### Statistical analysis

A linear model was utilized to compare average numbers (arithmetic means) of oocysts and sporozoites for each COM. The sporozoite data from mosquitoes which fed normal human serum (no TB antibodies) were used to build a zero-inflated negative binomial random effects model (ZINB model) which was similar to the model described previously [15]. Two more models (a negative binomial model and a zero-inflated Poisson model) were compared with the ZINB model by changing the parameters in the ZINB model: the zero-inflation parameter was fixed to zero for the negative binomial model, and the inverse dispersion parameter to infinity for the zero-inflated Poisson model. A non-standard likelihood ratio test was used to compare the two models against the ZINB model. Then the fitness of the ZINB model was evaluated using independent data set; mosquitoes fed with TB antibodies. To compare inhibition levels in oocyst and sporozoite intensities for each COM, Log Mean Ratio (LMR) was calculated for each stage of parasites as: Log_10_ [(average of oocysts or sporozoites in the control COM)/(average of oocysts or sporozoites in the test COM)]. Since %TRA is calculated as: 100 × [1 − (average in the test COM)/(average in the control COM)], it can be also expressed as; 100 × [1 − 1/(10^LMR^)]. The details of Bland–Altman analysis are shown in Additional file 2. All statistical tests were performed in R (version 3.4.1) or Prism 7 (GraphPad Software), and *P*-values ≤ 0.05 were considered significant.

## Results

**Fig. 1:**
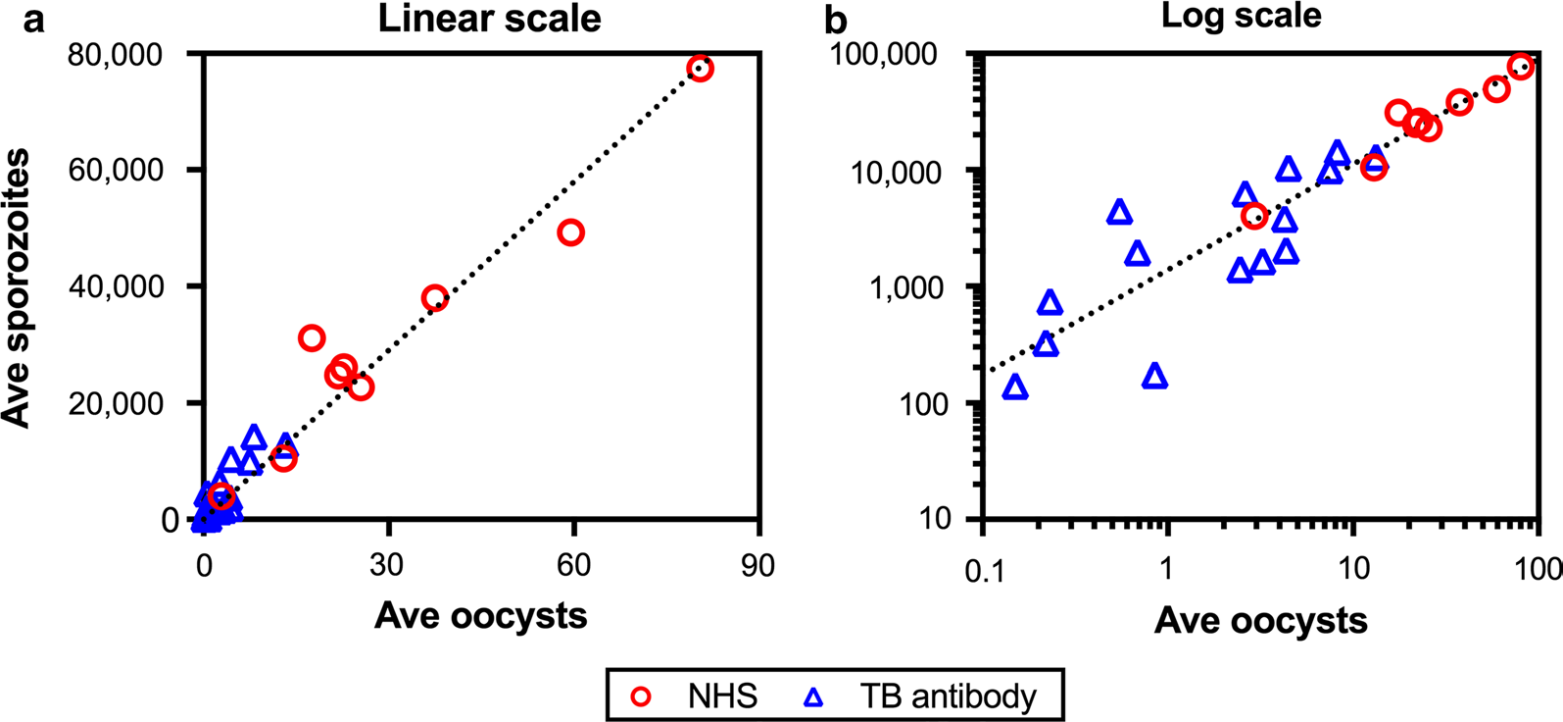
Correlation between average oocyst intensity and average sporozoite intensity. Arithmetic mean (Ave) of oocysts and sporozoites were calculated for each COM from 13 independent experiments. Nine COMs were fed with normal human serum (NHS), and 15 COMs with transmission-blocking antibodies (TB antibody). The same data are presented in either a linear-scale (**a**) or a Log-scale (**b**). The dotted line in each panel is the best-fit line for all data sets. A total of 532 mosquitoes were analyzed for oocysts, and 415 for sporozoites

We first evaluated whether there was any correlation between arithmetic mean (average) of oocyst intensity and that of sporozoite intensity, using data from 13 independent feeding experiments with 24 COMs including 947 mosquitoes. All oocyst and sporozoite counts in individual mosquitoes are shown in Additional file 1: Table S1. As shown in Fig. 1, there was a linear correlation between the two mean values, regardless of whether mosquitoes were fed with normal human serum (NHS) or with TB antibodies (*R*^2^ = 0.954). The intercept of linear model was not different from zero (the best estimate of 1741 with the 95% confidence interval (95% CI) of -200–3682), as expected (mosquito with zero oocysts should end up with zero sporozoites), and a likelihood ratio test showed that including the intercept did not improve the linear model. When the intercept term was excluded, the best estimate of slope was 966 (95% CI: 893–1038), meaning that one oocyst produced ~900–1000 salivary gland sporozoites, irrespective of whether the parasites were exposed to TB antibody or not before the oocyst formation.

**Fig. 2:**
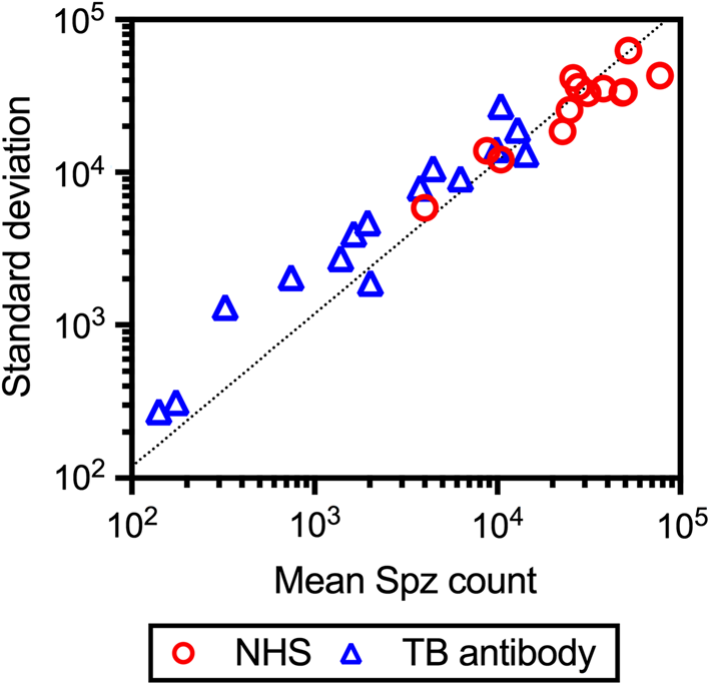
Generating a mathematical model for sporozoite data. For each COM, average and standard deviation of sporozoite values were calculated. The data from mosquitoes fed with NHS (13 COMs with 188 mosquitoes) were utilized to build a zero-inflated negative binomial (ZINB) model. The dotted line is the best-fit line calculated from the ZINB model, not the best-fit of all NHS and TB antibody data points. The fitness of the model to the mosquitoes fed with TB antibodies (15COMs with 227 mosquitoes) was *R*^2 ^= 0.707

To calculate the range of error in %TRA estimates at the sporozoite stage, we attempted to model the sporozoite data first. The data from mosquitoes fed with NHS (11 independent feeds with 13 COMs with 188 mosquitoes) were used to build the model. There was a strong correlation between average and standard deviation in each COM (Fig. 2) for sporozoite data, as shown in the oocyst data [11]. Therefore, we fitted a zero-inflated negative binomial (ZINB) model, as a ZINB model explains the oocyst data well [11, 15]. When two other models (a negative binomial model without zero-inflation and a zero-inflated Poisson model) were compared with the ZINB model, the ZINB model was supported (*P* < 0.0001 for both by likelihood ratio tests; Additional file 3). When the ZINB model was applied to an independent data set (mosquitoes were fed with TB antibodies; 15 COMs with 227 mosquitoes), the *R*^2^ was 0.707, indicating that it was reasonable to estimate the error range for sporozoite data using the ZINB model.

**Fig. 3:**
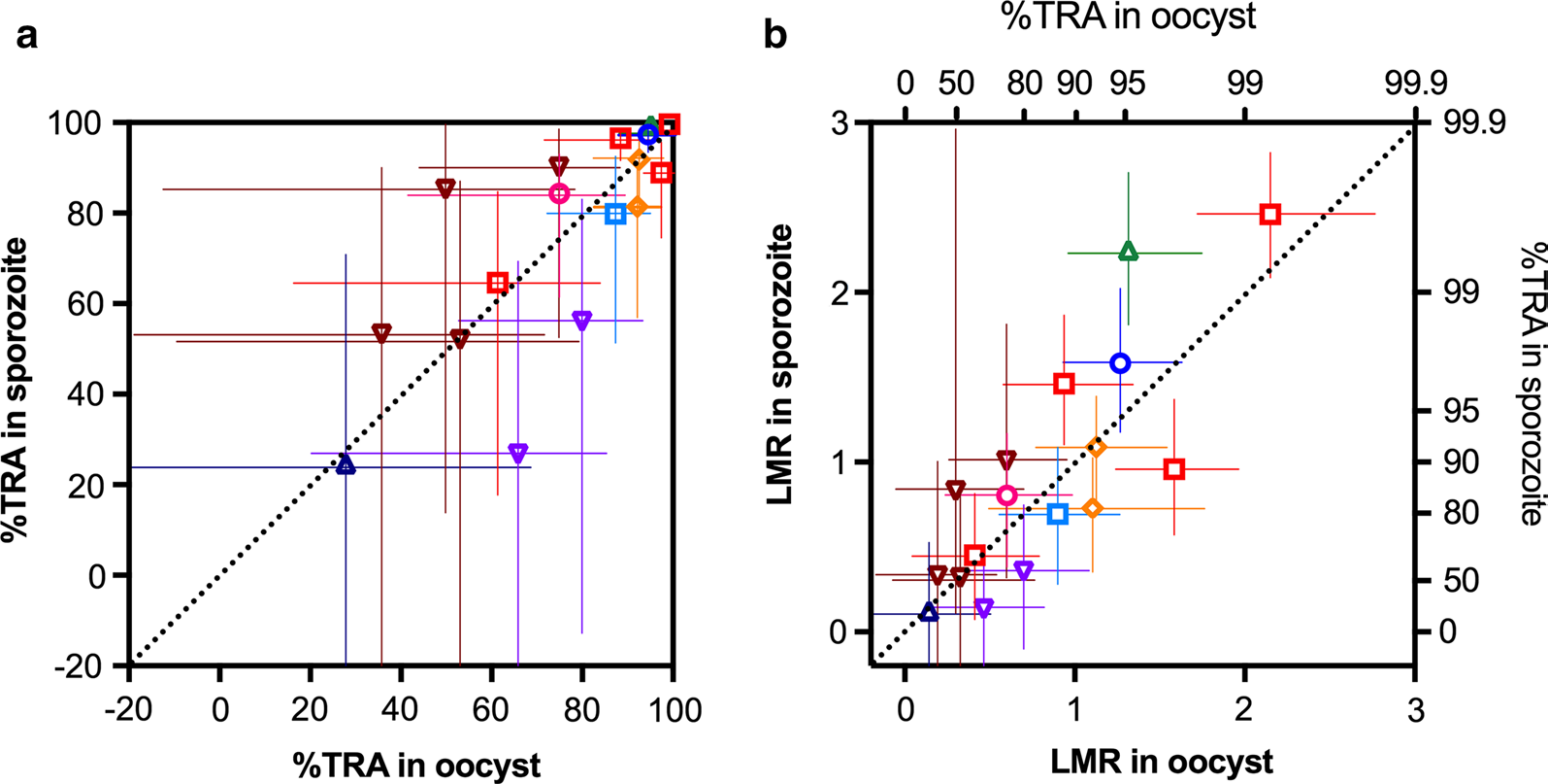
Concordance between inhibitions in oocysts and sporozoites. Inhibition levels in oocyst and sporozoite intensities were compared in the %TRA-scale (**a**) and the Log Mean Ratios (LMR)-scale (**b**). In **b**, the associated % inhibition (%TRA) value is shown on the right side of the y-axis or the top side of the x-axis. The best estimate and 95% CI for each test COM are shown. Points with the same symbol are from the same feed, and the dotted line is y = x. The red squires show the SMFA data with anti-Pfs48/45 antibody, and the other symbols with anti-Pfs25 antibodies

Inhibition levels in oocyst and sporozoite intensities were compared. The error in inhibition estimates were calculated from the oocyst-specific ZINB model [15] and the sporozoite-specific ZINB model described above. Similar to the oocyst data reported previously [11], the error range for the sporozoite data was also bigger at lower inhibition levels in a %TRA-scale (Fig. 3a). To address this issue, a transformation was applied to render the data on a Log Mean Ratio (LMR)-scale (Fig. 3b) and further analysis was performed using the LMR values. There was a linear correlation between LMR in oocysts and that in sporozoites, and the slope of the best-fit line was estimated as 1.080 (95% CI: 0.871–1.289). A likelihood ratio test showed that including the intercept did not improve the linear model, i.e. effectively zero. These results, along with the calculated random marginal agreement coefficient (RMAC [16]) of 0.796 (95% CI: 0.547–0.915) demonstrate that there was good agreement between LMR (conversion of %TRA) in oocysts and that in sporozoites, considering the error of measurements.

**Fig. 4:**
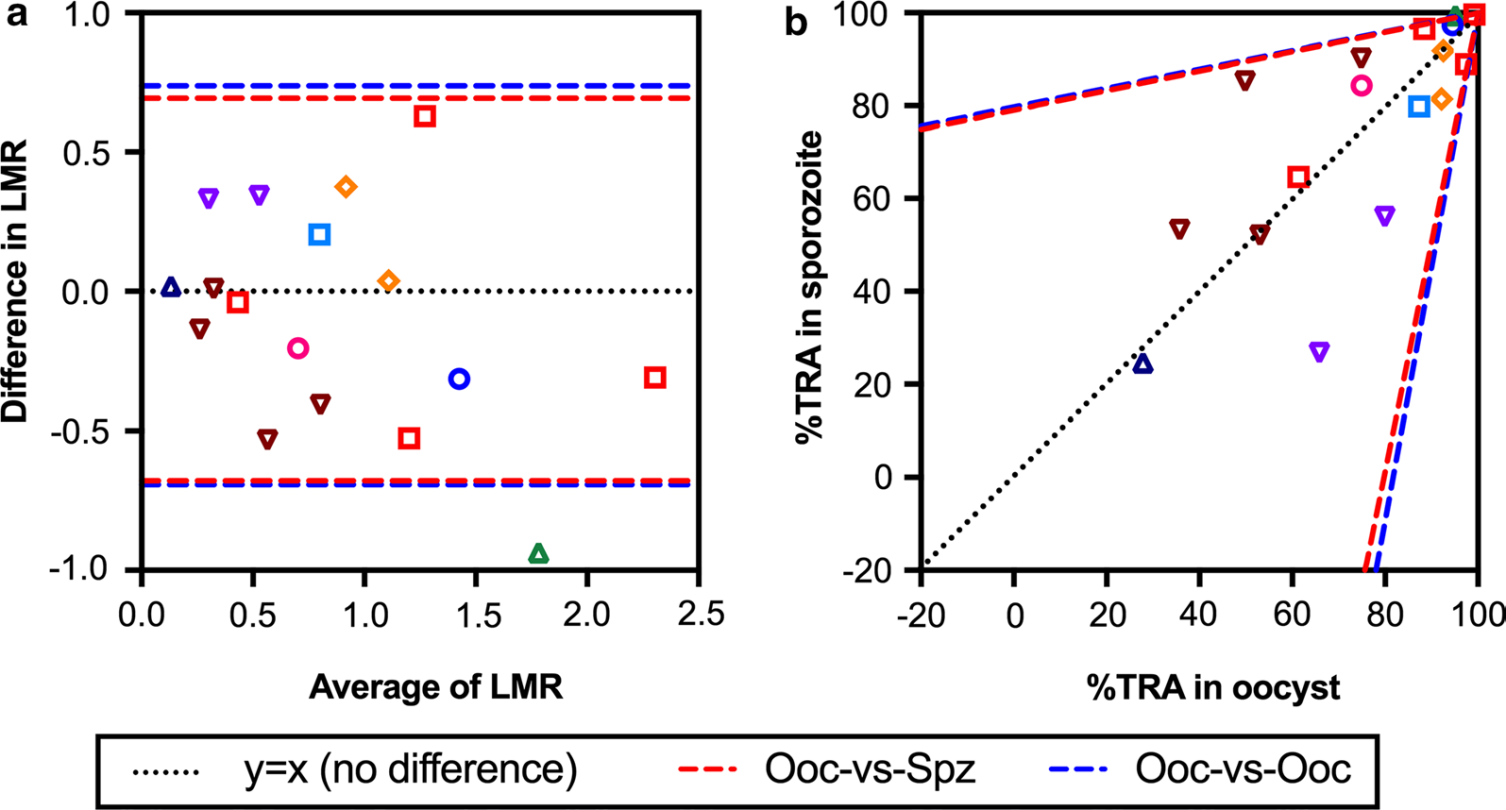
Bland–Altman prediction intervals for two measurements. The Bland–Altman prediction intervals (red and blue dotted lines) were calculated between (i) LMRs in oocysts and in sporozoites in the same COM (Ooc-vs-Spz; measured in this study), and (ii) LMRs in oocysts from two independent feeds, i.e. data from two different COMs (Ooc-vs-Ooc; reanalysis of previously reported SMFA data), using data where average LMR < 1.3 (average %TRA < 95%), as described in the supplemental material. **a** Bland–Altman plot of LMRs in oocysts and in sporozoites. **b** Each dot represents %TRA in oocysts and in sporozoites for each COM, and the prediction intervals were transformed to the %TRA-scale. The same symbols are used for the same COMs in Figs. 3 and 4

Finally, the agreement of LMRs in oocysts and in sporozoites was further evaluated by a Bland–Altman analysis (Fig. 4a and Additional file 2). When LMRs in oocysts and in sporozoites in the same COM were compared, the Bland–Altman prediction interval was calculated as − 0.679 to 0.693 (median difference of 0.007). To determine whether the agreement was better or worse than the agreement of LMRs in oocysts, but from two independent feeds, a reanalysis was done on a subset of previously reported SMFA data [15] (366 different samples tested in two independent feeds; a total of 732 LMR values). The prediction interval of LMR in oocysts from two different feeds was − 0.693 to 0.737 (median difference of 0.022, Fig. 4a), and the two intervals were practically the same in a %TRA-scale (Fig. 4b). The analyses suggest that to predict %TRA (or LMR) in sporozoites from the %TRA of oocysts in a single assay is not worse than predicting %TRA in oocysts in the second feed from that in the first feed.

## Discussion

In this study, we have shown for the first time that %TRA in oocysts can be directly converted to %TRA in sporozoites in *P. falciparum* SMFA, at least under these test conditions. In the preceding studies, a positive correlation between oocyst and sporozoite intensities has been observed with *P. falciparum* [5–7]. However, those studies were conducted without TB antibodies, and the numbers of mosquitoes used to determine sporozoite intensity were much smaller (16 to 46 mosquitoes per study) compared to this study, where a total of 536 mosquitoes were analyzed for their salivary gland sporozoite loads.

The previous experiments used *An. stephensi* mosquitoes, as this study, estimated median of 1250 salivary gland sporozoite per oocyst (inter-quartile range 313–2400, *n* = 46 mosquitoes) in one study [7] and geometric mean of 1361 sporozoite per oocyst (95% CI: 348–5322, *n* = 2) in another study [5]. Those numbers are very close to the number estimated from this study: 966 (95% CI: 893–1038) salivary gland sporozoite per oocyst. On the other hand, the *P. falciparum* studies using *An. gambiae* mosquitoes calculated slightly lower estimates; 663 sporozoites per oocyst (no error range was reported, *n* = 24) in one study [6] and 502 (95% CI: 217–1160, *n* = 5) in another study [5]. In case of other *Plasmodium* species, the estimated sporozoite numbers per oocyst were 508 (SD of 230 from 6 experiments; *An. dirus* or *An. minimus* mosquitoes) in *P. vivax* [8], and between 12 and 18 sporozoites per oocyst (no error range was reported, from 10 groups) for *P. berghei* in *An. stephensi* [10]. The difference between *P. falciparum* and *P. berghei*, in terms of number of salivary gland sporozoites produced from one oocyst, looks to be a true difference. However, since the numbers of mosquitoes analyzed in those studies were small, it is difficult to conclude whether there is a true difference between *P. falciparum* and *P. vivax*, or among different mosquito species. In addition, as described above, all those preceding studies were conducted without TB antibodies. An additional study is required to ascertain whether a strong concordance between %TRA in oocysts and in sporozoites holds true in other species of *Plasmodium* parasites and/or mosquitoes. However, we believe the results of this study will support designing such future studies.

Two values were used to express inhibition levels in this paper, %TRA and LMR. While %TRA has been used in the most of studies, since both oocyst and sporozoite intensity data follow negative binomial models (more specifically zero-inflated negative binomial models), lower inhibition values (higher oocyst or sporozoite intensity) have larger errors in the estimates (Fig. 3a). Therefore, at a lower inhibition level, LMR results could be easier to compare intuitively, because the transformation to render LMR estimates from TRA estimates makes the error range in LMR estimates appear more similar across inhibition levels (Fig. 3b). On the other hand, LMR might be misleading at a higher inhibition level. For example, a difference between 99 %TRA (LMR = 2) and 99.9 %TRA (LMR = 3) is very small biologically, but the difference in a LMR-scale is 1, which is the same difference between 0 %TRA (LMR = 0) and 90 %TRA (LMR = 1) in the scale. Considering the limitations for both readouts, the Bland–Altman analyses were performed with LMR values, but only used data where average %TRA was < 95% (or LMR < 1.3). If an average %TRA is greater than 95 % in a pair data set, the difference should be less than 10% point in a %TRA-scale (no sample can show > 100 %TRA by definition). Depending on the level of inhibition and scientific questions, a better readout should be selected for the analysis. While the Bland–Altman prediction interval for oocyst-to-sporozoite in the same feed was similar to that for oocyst-to-oocyst in two different feeds, both showed broad boundaries in a %TRA-scale (Fig. 4b). The analysis emphasizes the importance of reporting either %TRA or LMR values with their error ranges. To determine the error range, we used the oocyst-specific- ZINB model which we published previously [15] for %TRA in oocysts. For sporozoite data, we generated a new sporozoite-specific ZINB model in this study, and compared it with the other two nested models (Additional file 3). A recent study has shown that a beta-binomial (BB) model can be used to analyze oocyst data [17], and a similar BB model could explain sporozoite data as well. However, because BB models with non-linear predictors require the selection of an arbitrary maximum count, and those models are not nested within the ZINB, we did not compare ZINB and BB models in this study. It is an open question whether the BB or any other model can describe oocyst and sporozoite data better than our ZINB models.

In this study, only % inhibition in intensity (either %TRA or LMR), not % inhibition in prevalence of infected mosquitoes (% transmission-blocking activity, or %TBA), was utilized for the analysis. In theory, a proportion of mosquitoes with zero oocysts would be the same as the proportion of mosquitoes with zero sporozoites; i.e. %TBA in oocysts is the same as %TBA in sporozoites. Therefore, one might expect that %TBA readout is more straightforward. However, there are several disadvantages in the %TBA readouts. Firstly, it is practically very challenging to determine an accurate %TBA value for a group, especially when the infectivity is low, unless hundreds of mosquitoes per COM are examined. Indeed, a previous study has shown that a mosquito with no ruptured oocysts showed sporozoites in salivary glands, and another mosquito with ruptured oocyst displayed no sporozoites [7]. In our own study, we also observed 2 COMs, in which their averages of oocysts were very low (< 0.2 per mosquito), that demonstrated averages of “zero” sporozoites in 14 or 16 mosquitoes dissected (those data were not included in this analysis, as it is difficult to estimate an error range for zero). The second issue in the %TBA readout is robustness. In case of oocyst counts, we have shown that %TBA is a function of %TRA and the mean oocysts in the control group [15], while %TRA is independent from the mean control oocyst. To state this another way, %TBA values from different experiments with different mean control oocysts cannot be directly compared unless adjusted for the mean control (standardized %TBA, as proposed previously [15]), whereas %TRA values do not require such adjustment. This study did not generate enough data to validate the correlation among %TRA, %TBA and control mean intensity in sporozoites; however, it is intuitively reasonable to argue that to bring down the sporozoite number from 70,000 to zero is harder than from 140 to zero. Because targeting the control mean of oocysts or sporozoites in a given feeding experiment is practically impossible at this moment, %TRA is considered a more robust readout. The third point is predictability of infection from the infected mosquito to a human host. A recent study of controlled human malaria infection (CHMI) *via* mosquito bites has shown that a higher dose of salivary gland *P. falciparum* sporozoites significantly correlates with higher infectivity in humans challenged [18]. In addition, another CHMI study with intravenous injection of cryopreserved sporozoites (50–3200 per inoculation) has also shown a significant positive association between the dose of inoculation and infectivity in humans [19]. On the other hand, a CHMI study by mosquito bites using much higher doses of sporozoites (mean of 78,415 sporozoites per mosquito) than seen in wild-caught mosquitoes (median or geometric mean of around 800 to 6000 [20, 21]) showed no correlation between sporozoite loads and time to parasitemia [22]. Taken together, for each infected mosquito bite, it is reasonable to assume that the probability to infect humans depends on the sporozoite intensity in salivary glands, rather than any number of sporozoites, when the sporozoite intensity is within the range seen in the field.

There are several caveats if one wants to incorporate the strong concordance observed in this study to predict the efficacy of TBV in the field. While the majority of TB data in this paper were generated using anti-Pfs25 antibodies (which block the post-fertilization step), some used anti-Pfs48/45 mAb (which block the pre-fertilization step). If there is a minor difference between the two types of antibodies (while it is unlikely to affect the oocyst to sporozoite transition), this study does not have an enough power to detect a small difference. In addition, this study specifically focused on the effect of TB antibodies ingested with blood meals. If a drug, a transgenic mosquito, or other intervention, interferes with the later stage of parasite development in mosquitoes, the correlation between oocyst and sporozoite inhibitions needs to be reevaluated. One of the major differences between the artificial SMFA and natural infection in the field is parasite intensity. The mathematical models have shown that it is difficult to determine %TRA accurately when the oocyst intensity is low, like the level observed in the field, unless hundreds of mosquitoes are analyzed [15, 23]. Since sporozoite data are also explained by a similar ZINB model, it is reasonable to predict that it is true for sporozoites as well. If the prediction is right, determining the concordance between the two sets of %TRA values in the field (or in SMFA performed with low parasite density to mimic the field situation) is practically very challenging. Further complications that can change the dynamics of these assays are: if the ratio of non-ruptured (which do not produce sporozoites) and ruptured (produce sporozoites) oocysts, and/or the mortality of infected mosquitoes (whether an infected mosquito can survive for two weeks after gametocyte ingestion) could be affected by the parasite intensity, especially at the low (field-like) level of intensity. If one wants to prove (or disprove) whether the strong concordance still holds at low parasite intensity, a targeted (and likely very large) study is required.

## Conclusions

The present study covered a large range of mean oocyst (0.2–80.5) and sporozoite (141–77,417) intensities and showed strong concordance between %TRA in oocysts and %TRA in sporozoites for the first time in *P. falciparum* SMFA. The strong concordance justifies the usage of %TRA in oocysts, instead of %TRA in sporozoites, which is practically more difficult and requires an extra week for the assay. This study will further assist modeling of TBV efficacy from SMFA results.

## **Additional files**

**Additional file 1: Table S1.** Oocyst and sporozoite numbers used for the analysis. Details of each feeding condition and resulting oocyst and sporozoite numbers in each mosquito are shown (XLSX 29 kb).

**Additional file 2.** Details of Bland–Altman analysis (Fig. 4) for both Ooc-vs-Spz and Ooc-vs-Ooc are shown (pdf 382 kp).

**Additional file 3.** Details of zero-inflation negative binomial model fit of sporozoite data (pdf 149 kp).

## Abbreviations

CHMI: controlled human malaria infection
COM: container of mosquitoes
NHS: normal human serum
LMR: Log Mean Ratio
mAb: monoclonal antibody
%TBA: % transmission-blocking activity
%TRA: % transmission reducing activity
SMFA: standard membrane-feeding assay
TB: transmission-blocking
TBV: transmission-blocking vaccine
