# Supplementary material for: Strong concordance between percent inhibition in oocyst and sporozoite intensities in a Plasmodium falciparum standard membrane-feeding assay
Source: Parasit Vectors. 2019 May 6;12:206. doi: 10.1186/s13071-019-3470-3 (PMC6501457; doi:10.1186/s13071-019-3470-3)
Supplement: Supplementary file 2 — Additional file 2. Details of Bland–Altman analysis (Fig. 4) for both Ooc-vs-Spz and Ooc-vs-Ooc are shown (pdf 382 kp). [file 13071_2019_3470_MOESM2_ESM.pdf]

Additional File 2

This supplemental document is a record of the attempts to deal with the nuances that come with trying to explore the agreement between TRA readouts on oocyst data and TRA readouts on sporozoite data. `logrho` can be used interchangeably with the main text's "LMR", as can "TRA" be used interchangeably with the main text's "%TRA".

Of 20 samples, three ("94_1_2", "109_2_16", "104_1_2") are presented initially but are quickly excluded. All three of these samples had 0 sporozoites across all mosquitoes. In addition, "94_1_2" and "104_1_2" had > 99% TRAs in the oocysts, and "109_2_16" had a mean control of 0.66 which hampered accurate TRA estimation. The software and method of calculating the TRA and the confidence intervals in the tables that follow below are from Swihart, Fay & Miura (Journal of the American Statistical Association 2018).

Quick definitions:

- tra.spz: The transmission reducing activity in the sporozoite domain, that is 100*(1-(mean sporozoite counts of treated)/(mean sporozoite counts of control))
- tra.spz.95L: The lower bound of the 95% confidence interval for tra.spz
- tra.spz.95U: The upper bound of the 95% confidence interval for tra.spz

And similarly for oocysts:

- tra.ooc: The transmission reducing activity in the oocyst domain, that is 100*(1-(mean oocyst counts of treated)/(mean oocyst counts of control))
- tra.ooc.95L: The lower bound of the 95% confidence interval for tra.ooc
- tra.ooc.95U: The upper bound of the 95% confidence interval for tra.ooc

Table S1: TRAs and 95%CI for Sporozites, sorted by `tra.spz`

```
##      feed   sample   tra.spz tra.spz.95L tra.spz.95U
##  1: 109_2 109_2_16 100.00000          NA          NA
##  2:  94_1   94_1_2 100.00000          NA          NA
##  3: 104_1  104_1_2 100.00000          NA          NA
##  4: 108_1 108_1_19  99.65238    99.20148    99.84791
##  5: 108_2  108_2_2  99.43723    98.38537    99.80760
##  6: 107_2  107_2_2  97.38428    93.49134    99.09021
##  7: 108_1 108_1_21  96.55686    91.88452    98.61105
##  8: 122_2 122_2_17  91.87500    81.47509    95.97320
##  9: 104_1  104_1_5  90.16393    53.38873    98.48651
## 10: 108_1 108_1_20  88.97109    74.53508    95.84985
## 11: 104_1  104_1_4  85.24590    19.28967    99.63286
## 12:  96_2   96_2_9  84.34263    59.05632    93.26222
## 13: 122_2 122_2_18  81.40625    58.28473    90.79831
## 14:  97_2   97_2_2  79.85100    50.16666    92.70202
## 15: 108_1 108_1_22  64.67104    16.23005    83.68815
## 16: 116_1  116_1_9  56.02339   -21.59210    82.67493
## 17: 104_1  104_1_3  53.27869  -106.78993    89.63536
## 18: 104_1  104_1_6  52.04918  -100.05264    86.54731
## 19: 116_1 116_1_10  26.72859   -88.66527    70.21615
## 20:  98_2  98_2_13  24.68811   -81.33509    72.01239
```

Table S2: TRAs and 95%CI for *Oocysts*, sorted by `tra.ooc`

```
##      feed   sample  tra.ooc tra.ooc.95L tra.ooc.95U
##  1:  94_1   94_1_2 99.60000   98.333333    99.85337
##  2: 104_1  104_1_2 99.32050   97.752809    99.87923
##  3: 108_1 108_1_19 99.29245   98.014440    99.88479
##  4: 108_1 108_1_20 97.40566   92.307692    99.57983
##  5: 108_2  108_2_2 95.15670   87.654321    99.02913
##  6: 107_2  107_2_2 94.59291   86.718750    98.46939
##  7: 122_2 122_2_17 92.53659   80.097561    98.07147
##  8: 122_2 122_2_18 92.15385   79.972280    97.18776
##  9: 108_1 108_1_21 88.44340   72.656250    95.92760
## 10:  97_2   97_2_2 87.39496   70.957230    94.24579
## 11: 116_1  116_1_9 79.99339   47.619048    92.69103
## 12:  96_2   96_2_9 74.90347   40.000000    90.16949
## 13: 104_1  104_1_5 74.85844   43.764706    89.36170
## 14: 109_2 109_2_16 71.04762   32.480455    87.99806
## 15: 116_1 116_1_10 65.80688   22.969188    86.52186
## 16: 108_1 108_1_22 61.32075   10.256410    83.78378
## 17: 104_1  104_1_6 53.00113  -13.265306    80.66465
## 18: 104_1  104_1_4 49.83012   -9.384683    77.58953
## 19: 104_1  104_1_3 35.78709  -44.687500    71.37662
## 20:  98_2  98_2_13 27.84314  -64.179104    70.69486
```

For the 17 points (we exclude "94_1_2", "109_2_16", "104_1_2"), on the LMR scale, we conduct a Bland-Altman analysis where the mean of measurements (x-axis) is plotted with the difference (y-axis) along with the mean difference line flanked by the upper and lower limits of agreement referred to as the "bounds of agreement" (see The Lancet Paper by Bland and Altman "Statistical Methods for Assessing Agreement Between Two Methods of Clinical Measurement" for more details.)

Figure S1: Bland-Altman LMR scale of 17 points

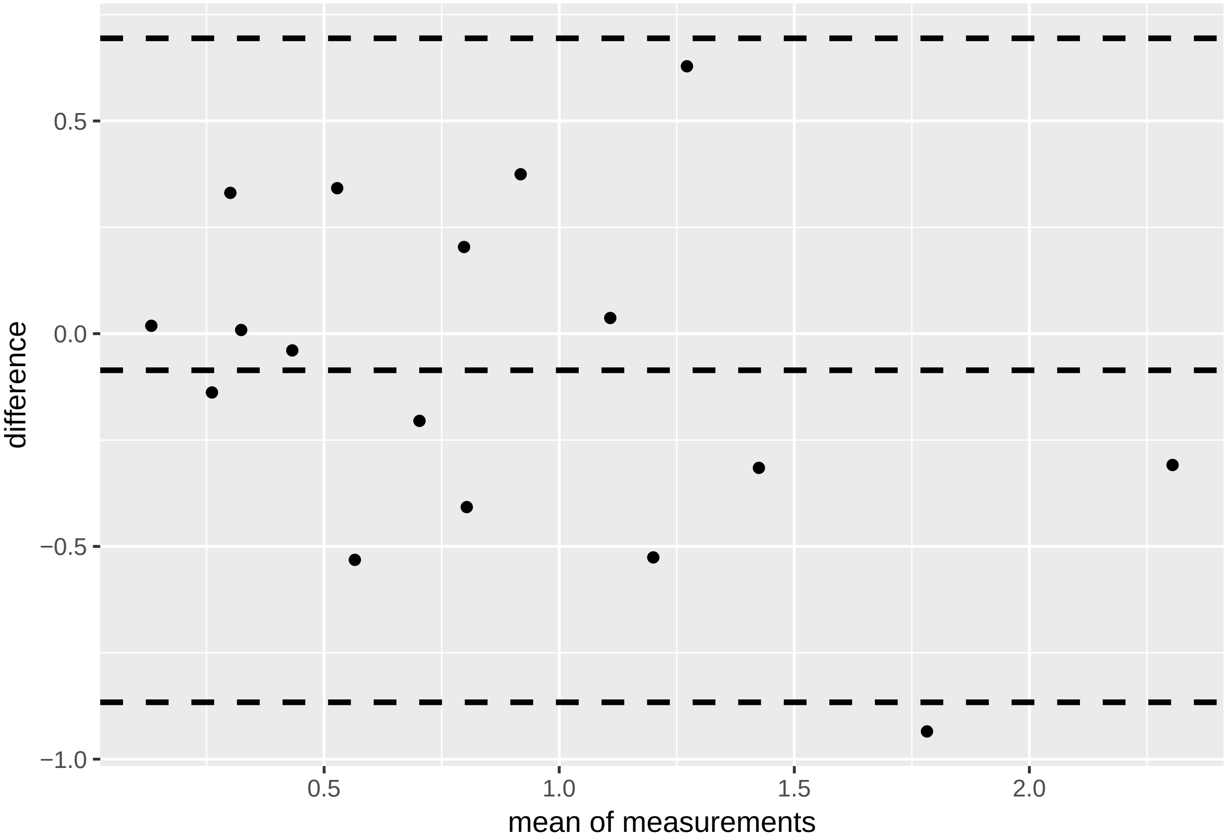

All (except one point) are between the lines of agreement. One might think this is a strong case for agreement, but that would be an incorrect interpretation of the plot. The bounds of agreement (the lowest line and highest line) are

```
## lower.limit upper.limit
##  -0.8663025   0.6943020
```

which corresponds to a TRAs of

```
100*(1-10^(-(-0.87)))
```

```
## [1] -641.3102
```

```
100*(1-10^(-( 0.69)))
```

```
## [1] 79.58262
```

The transformed bounds of agreement on the difference are (-641, 79.6) – not very informative for TRA measures.

We decided to explore further and try to discover why this range is so large.

Our first thought was to take the (-0.87, 0.69) Bland-Altman (BA) bounds on the LMR scale for the 17 points and transform them to be bounds on the `tra.spz` as a function of `tra.ooc` so that we could the have equivalent of the Bland-Altman bounds on the `tra.spz` vs `tra.ooc` plot (Figure S2).

Figure S2: TRA-spz vs TRA-ooc with transformed BA bounds

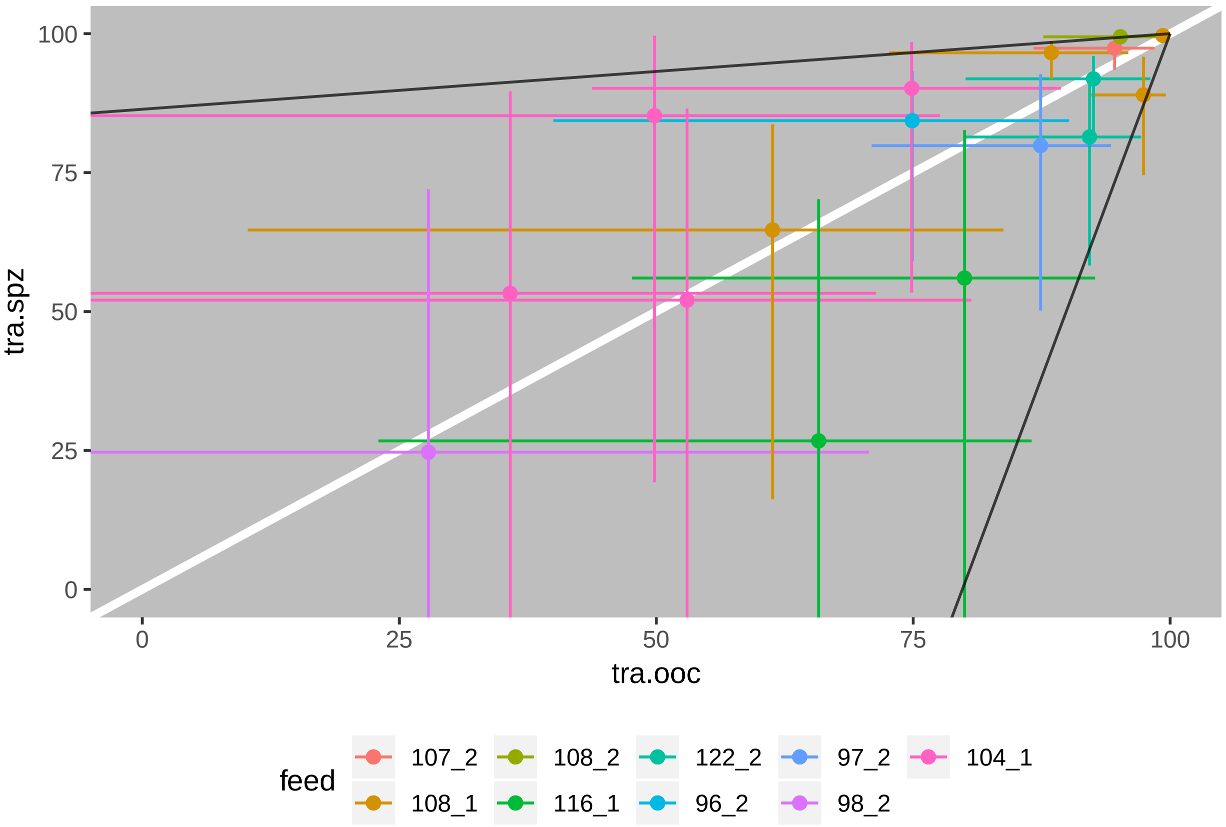

Figure S2 indicates that if a `tra.ooc` is approximately 80 then the prediction interval for `tra.spz` is (1.07, 97.28).

We want to compare BA bounds between Ooc-Spz pairs and Ooc-Ooc pairs to determine whether the agreement was better or worse than the agreement of LMRs in oocysts (but from two independent feeds). A reanalysis was done on a subset of previously reported SMFA data (see reference [15] in main text). For our reanalysis, we look at 2 feeds of 79 sample sets. Within a sample set, the two feeds each had the same number of test COMs and 1 control COM (i.e. container of mosquitoes). However, the number of test COMs did vary across sample sets. Thus, we had 2*79=158 control COMS (3,221 mosquitoes) and 994 test COMs (20,244 mosquitoes) for an average of about 20 mosquitoes per COM. Ultimately we have 497 pairs of LMR values in this analysis.

Figure S3: Bland-Altman for the repeated SMFA (LMR scale, shown without bounds):

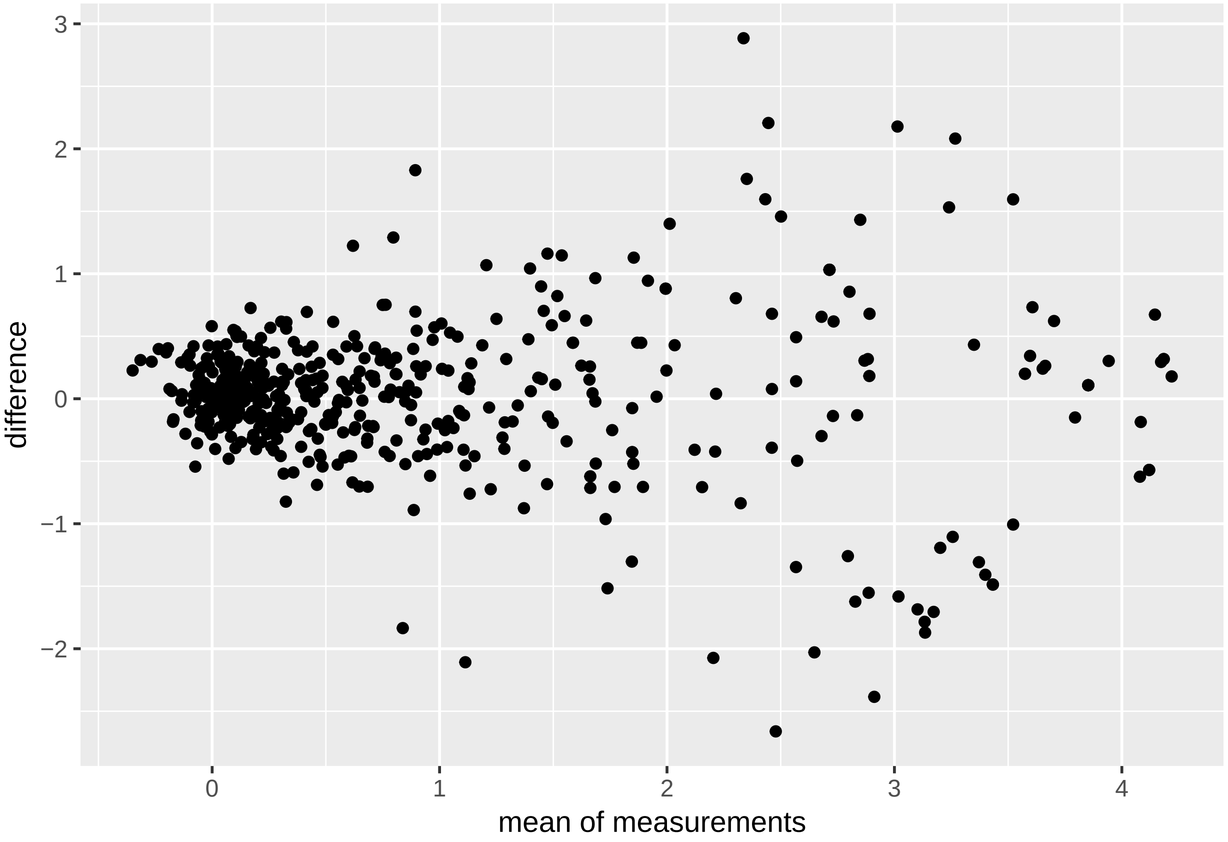

While %TRA has been used in the most of studies, since both oocyst and sporozoite intensity data appear to follow negative binomial models (more specifically zero-inflated negative binomial models), lower inhibition values (higher oocyst or sporozoite intensity) have larger errors in the estimates (Fig. 3A of the main text). Therefore, at a lower inhibition level, LMR results could be easier to compare intuitively, because the error range in LMR estimate is less affected by the inhibition level (Fig. 3B of the main text). On the other hand, LMR might be misleading at a higher inhibition level. For example, a difference between 99 %TRA (LMR=2) and 99.9 %TRA (LMR=3) is very small biologically, but the difference in a LMR-scale is 1, which is the same difference between 0 %TRA (LMR=0) and 90 %TRA (LMR=1) in the scale. Considering the limitations for both readouts, the Bland-Altman analyses were performed with LMR values, but only used data where average %TRA was < 95% (or LMR < 1.3). Restricting to the average LMR<1.3 reduced the data to 2 feeds of 76 (of 79) sample sets. Thus, we had 2*76=152 control COMS (3,101 mosquitoes) and 732 test COMs (14,858 mosquitoes) for an average of about 20 mosquitoes per COM. Ultimately we have 366 pairs for LMR values in this analysis.

Of note, several points demonstrated mean LMR <0 in Fig S3. We think the negative LMR are likely due to the variability of the assay, not "enhancement of parasite transmission". Indeed, almost all such negative data are not significantly difference from no inhibition (zero %TRA) in each feed based on our zero-inflated negative binomial model (data not shown). Therefore, to evaluate the random variability of the assay more accurately, we decided to include the negative LMR values in the analysis.

Figure S4: Bland-Altman (LMR scale) for the repeated SMFA (all Ooc-Ooc in black, ave LMR < 1.3 in red):

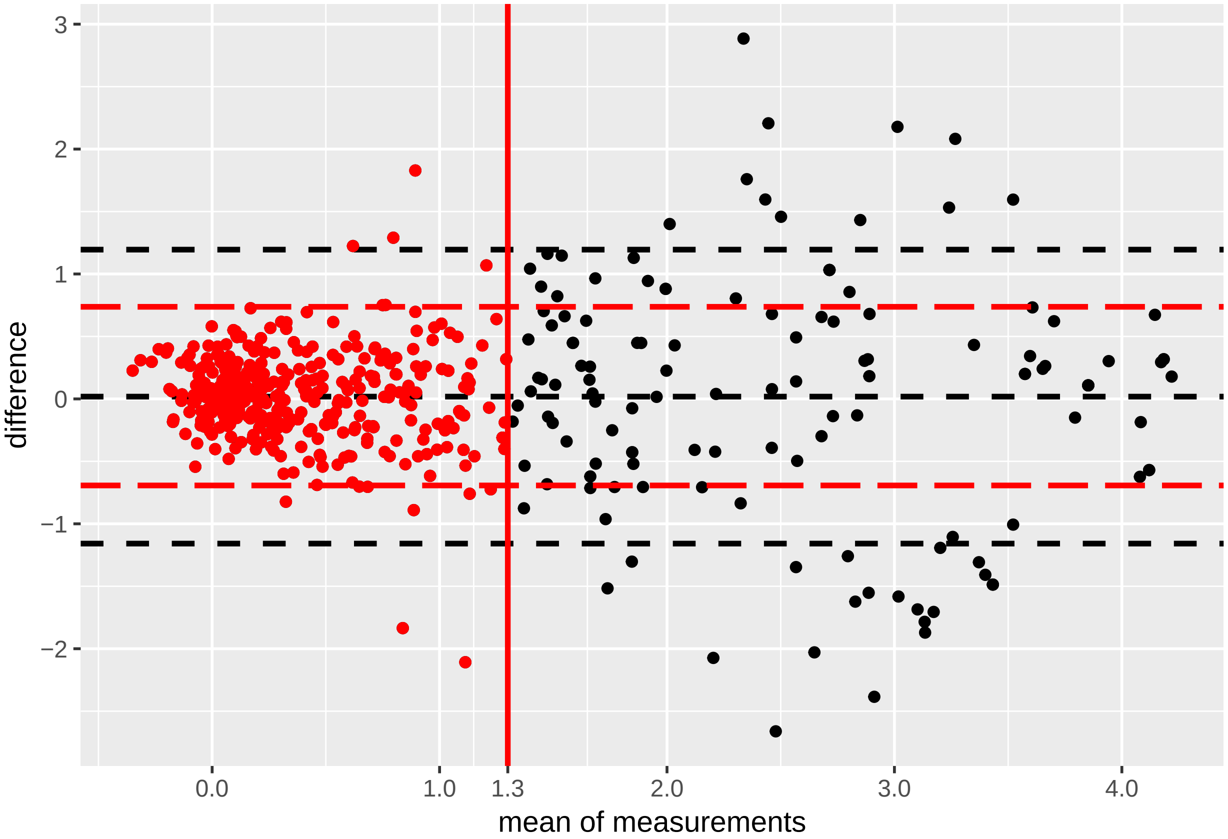

Given the reasonable reduction of outlying points on the Bland-Altman plot in Figure S4, we opt to re-do the Bland-Altman on the original ooc-vs-spz dataset, restricted to LMR < 1.3 (which removes three points), we observe that the original BA bounds were (-0.866,0.694) and removing the three points gives (-0.679,0.693) and display this in Figure S5:

Figure S5: Bland-Altman (LMR scale) for the Spz-Ooc (all Spz-Ooc in black, ave LMR < 1.3 in red):

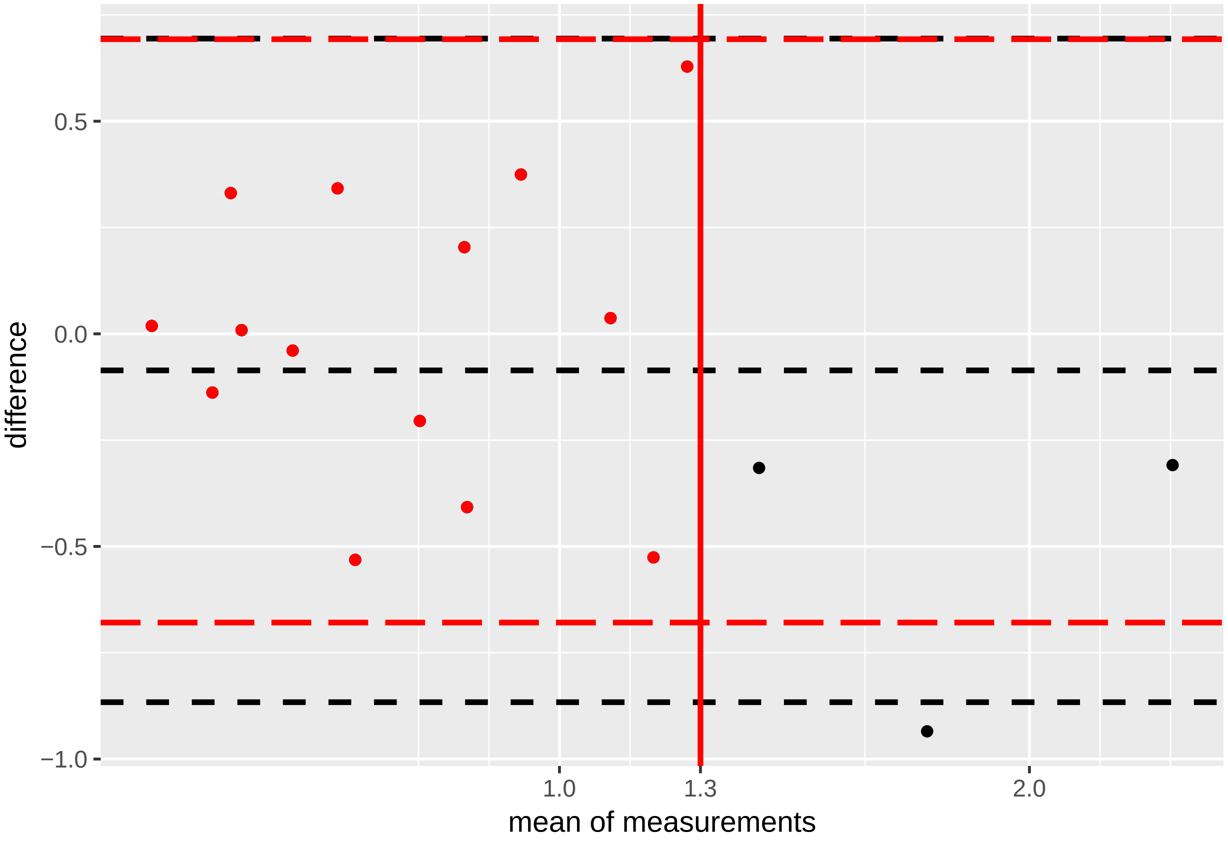

Below in Figure S6 the transformed plot is displayed with both the ooc-vs-spz data and repeated SMFA bounds calculated on the data with average LMR < 1.3:

Figure S6: TRA-spz vs TRA-ooc with transformed BA bounds

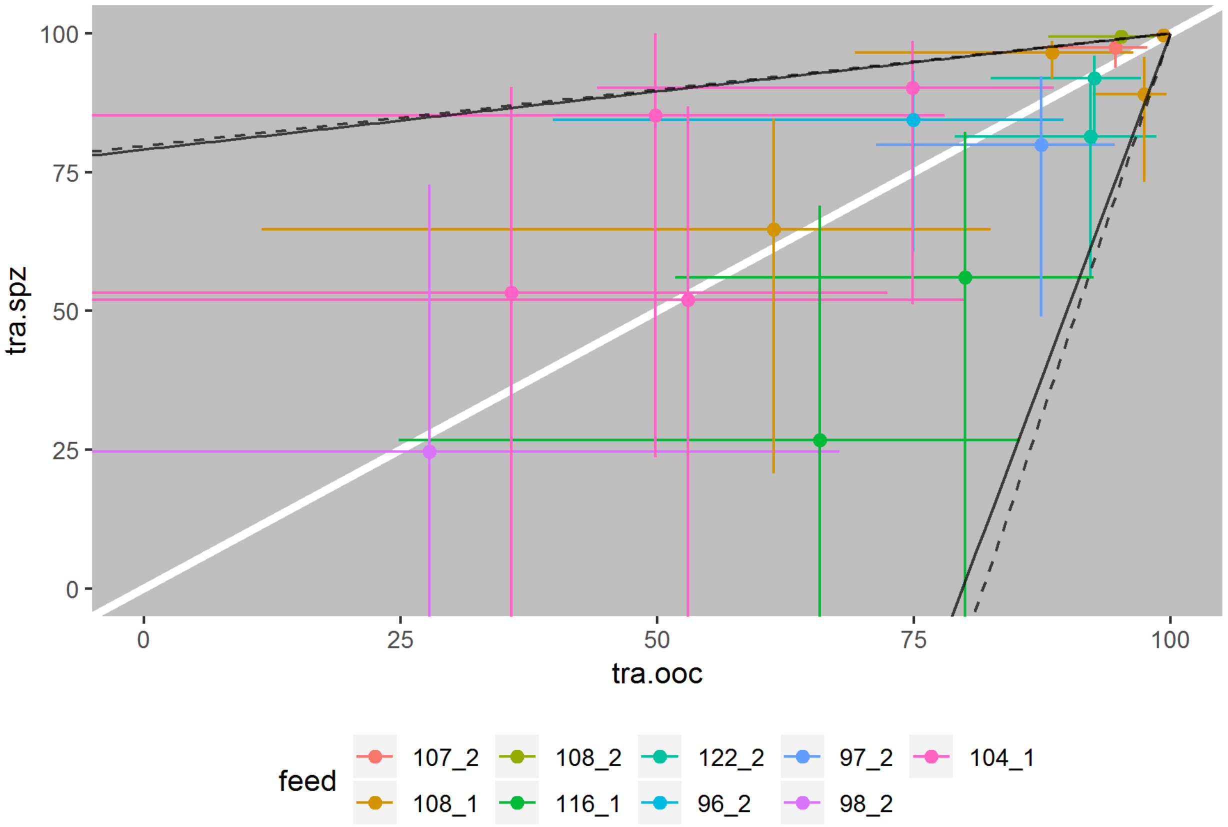

Figure S6 is picked for publication (Figure 4B in manuscript; Figure 4A in the manuscript shows similar info). Eliminating LMRs > 1.3 does not change the practical implications of agreement but allows Bland-Altman to calculate bounds that are not overly penalized on the LMR scale. The `figure` immediately above shows that tra.spz agrees with tra.ooc just as well as tra.ooc replicates agree with tra.ooc

The fact that the bounds are so poor speaks to the difficulty in replicating SMFAs. This is well known and the reason why random effect modeling is needed. (See Swihart, Fay, Miura in JASA 2018).

One key observation is that an LMR > 1.3 corresponds to a TRA of 95. There are 3 points of the 17 that are above a mean LMR of 1.3. For practical purposes, if a pair of TRAs averaged to 95, they would be thought to be highly agreeing in treatment effect (the biggest range would be for a TRA of 100 being paired with a TRA of 90). We also decided a similar notion of "highly agreeing" could be said that if both TRAs were above 90. We then realized that while the LMR transformation was good for calculating Bland-Altman statistics, the interpretability and intuition laid with the TRA scale, so we wanted to try Bland-Altman on TRA scale as well.

Even though we selected the LMR < 1.3 option for the published manuscript, given the observations in the paragraph above, we decided to explore Bland-Altman on the TRA scale. For the sake of transparency, it is listed below. It is NOT included in the main text and appears solely in this document.

Bland-Altman on the TRA scale

The BA boundary lines in Figure S7 are:

```
## lower.limit  mean.diffs upper.limit
## -32.4103158  -0.1179195  32.1744768
```

Indicating that the bounds of agreement on the difference using the TRA scale is about -32 to 32.

Figure S7: Bland-Altman TRA scale of 17 points

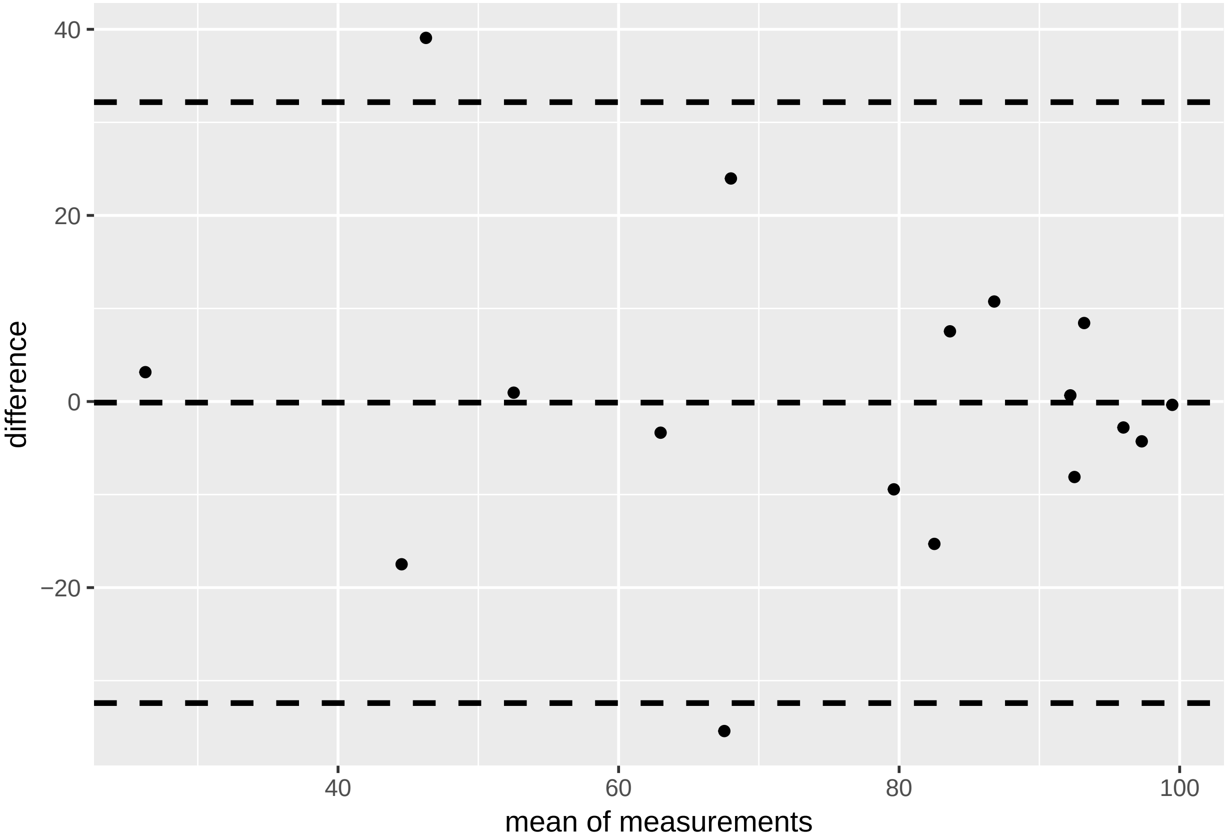

If we take the "bounds" from plot above and put on the TRA-ooc vs. TRA-spz plot, we get the following – note the parallel lines:

Figure S8: TRA-spz vs TRA-ooc with transformed BA bounds from Fig S7

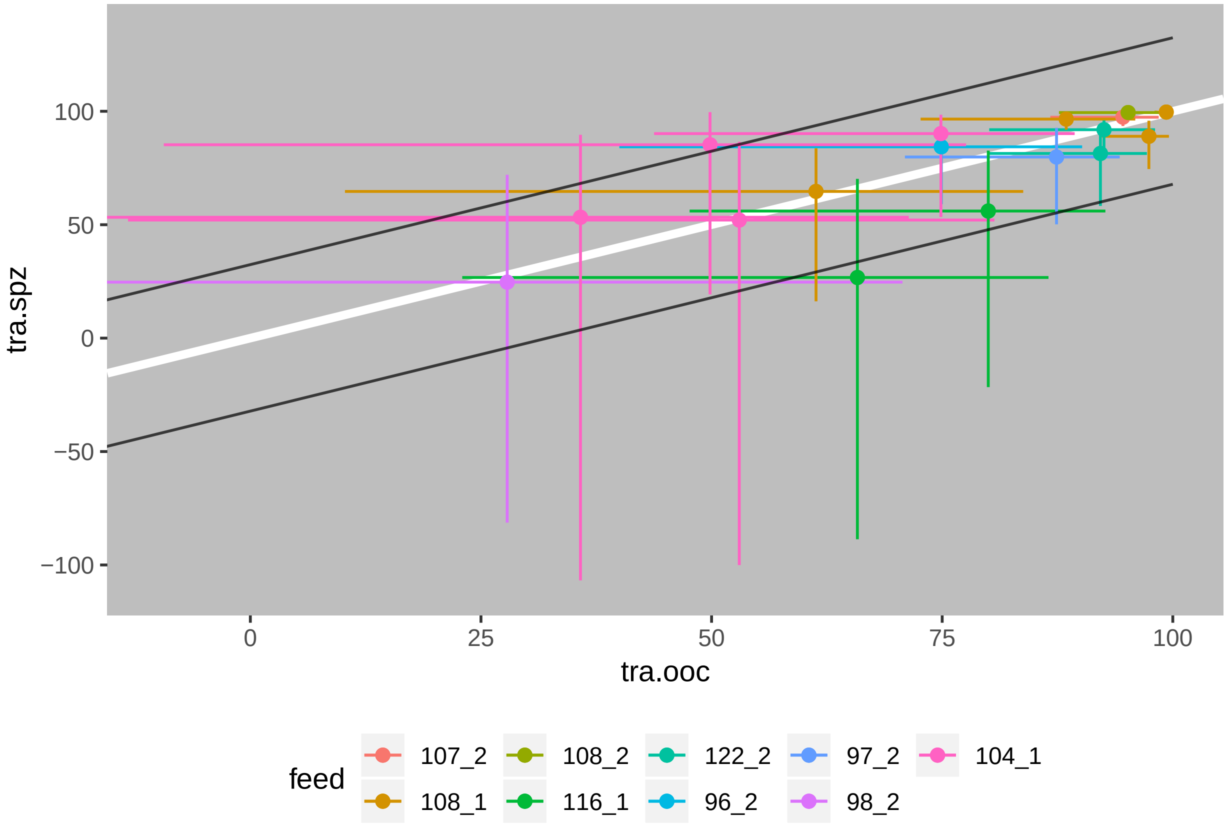

Now we do the same for the Repeated SMFA Bland-Altman plot on TRA scale.

The bounds are:

```
## lower.limit  mean.diffs upper.limit
##   -68.94038     4.74484    78.43006
```

Figure S9: Bland-Altman for the repeated SMFA (TRA scale)

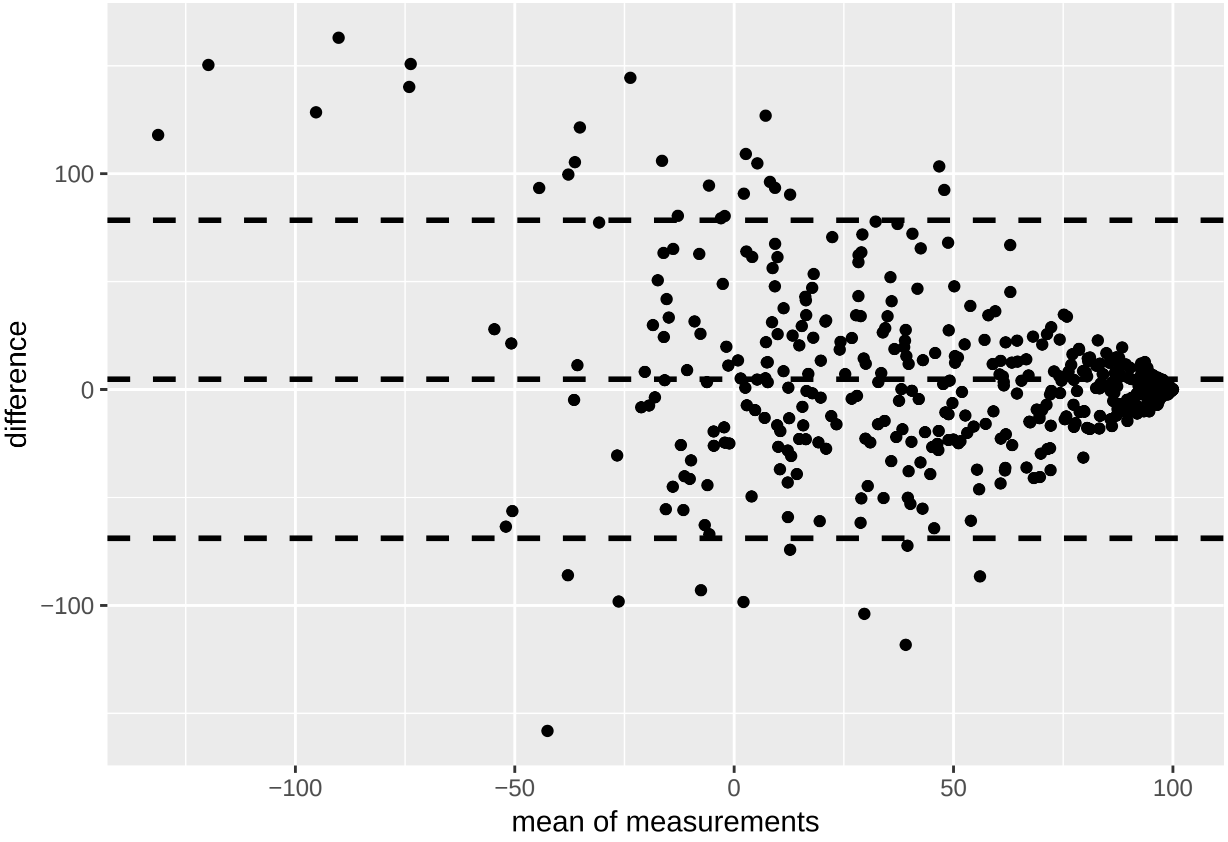

And we can add the new bounds (dashed-lines) to the tra.spz vs tra.ooc plot like so:

Figure S10: TRA-spz vs TRA-ooc with transformed BA bounds from Figs S7 and S9

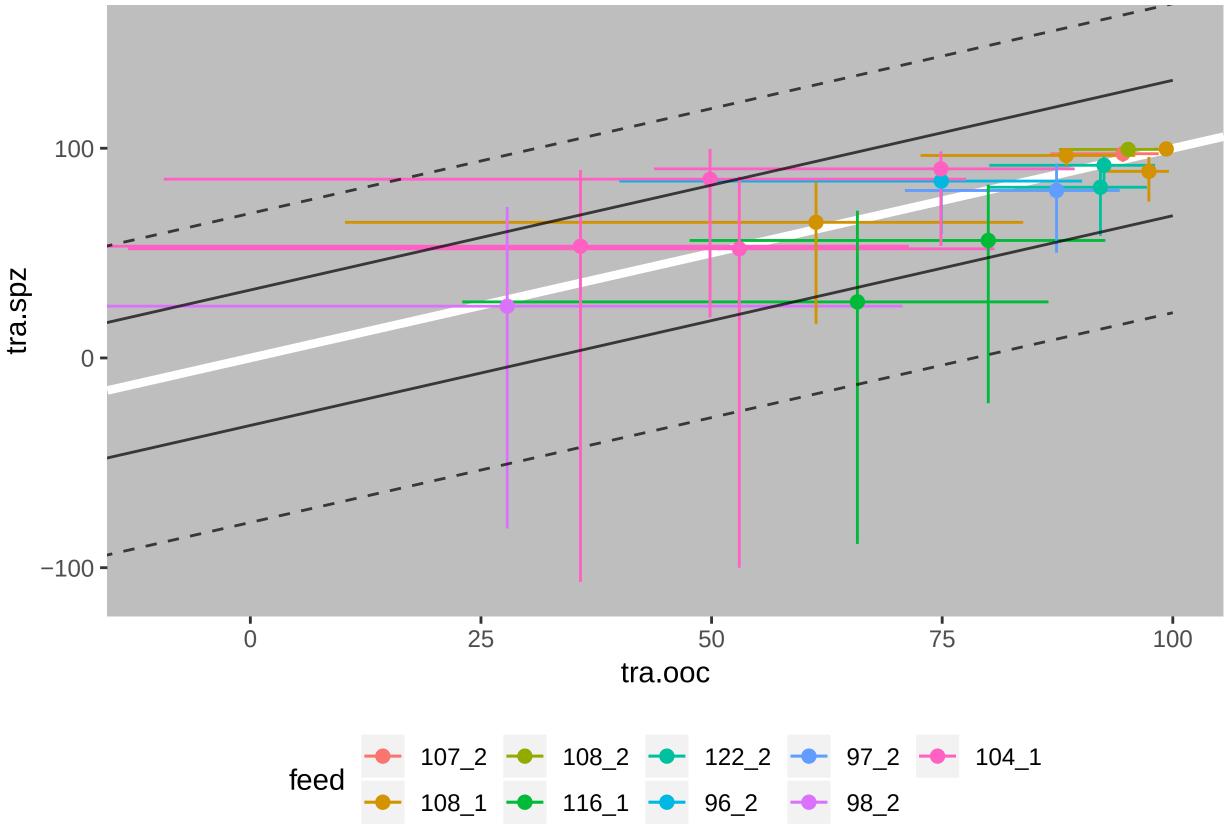

. . . Or we can add them to the Bland-Altman plot. The outermost ones are for the repeated SMFA data on the TRA scale:

Figure S11: Bland-Altman TRA scale of 17 points with bounds from Fig S10

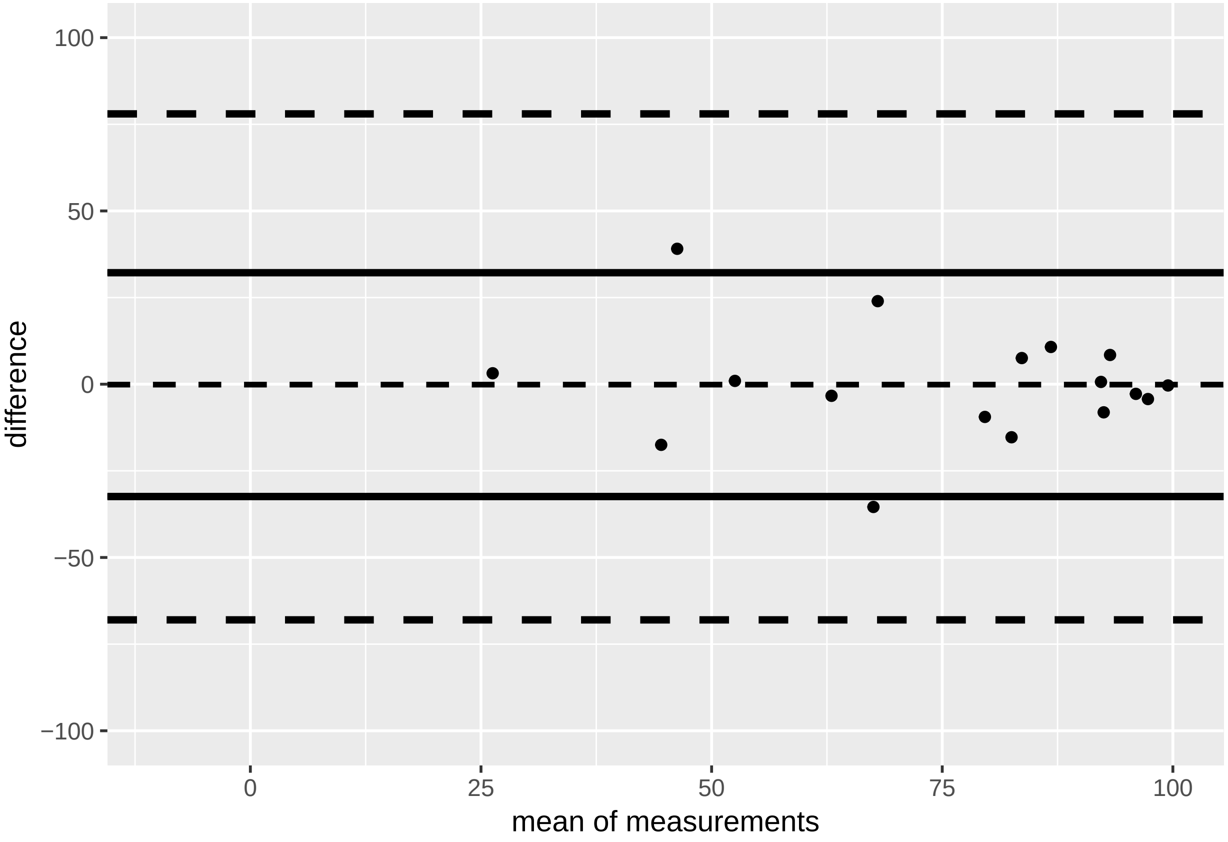

The Bland-Altman plots of the TRA scale were not selected for publication as the primary graphic because there were clear associations between mean and difference (i.e., lower mean values had higher difference) (see Fig S7 and Fig S9) on TRA scale.
