# Supplementary material for: Strong concordance between percent inhibition in oocyst and sporozoite intensities in a Plasmodium falciparum standard membrane-feeding assay
Source: Parasit Vectors. 2019 May 6;12:206. doi: 10.1186/s13071-019-3470-3 (PMC6501457; doi:10.1186/s13071-019-3470-3)
Supplement: Supplementary file 3 — Additional file 3. Details of zero-inflation negative binomial model fit of sporozoite data (pdf 149 kp). [file 13071_2019_3470_MOESM3_ESM.pdf]

Additional File 3

This supplemental document describes the detail of model fit for the sporozoite count results, and compares the fitness of three models (zero-inflated negative binomial, negative binomial, and zero-inflated Poisson). In addition it outlines how an R^2 statistic is computed.

We fitted a zero-inflated negative binomial model (zinb) to the controls of the sporozite (Spz) data (above) with the following R command:

```
library(glmmTMB)
spz.fit.zinb <-glmmTMB(spz ~ 1+(1|feed)+(1|pint),
                       family="nbinom2",
                       ziformula=~1,
                       data=spz.supp ## controls only
)
print(spz.fit.zinb)
```

```
## Formula:          spz ~ 1 + (1 | feed) + (1 | pint)
## Zero inflation:       ~1
## Data: spz.supp
##       AIC       BIC    logLik  df.resid
##  3951.087  3967.270 -1970.544       183
## Random-effects (co)variances:
##
## Conditional model:
##  Groups Name        Std.Dev.
##  feed   (Intercept) 0.5464998
##  pint   (Intercept) 0.0004577
##
## Number of obs: 188 / Conditional model: feed, 11; pint, 13
##
## Overdispersion parameter for nbinom2 family (): 0.936
##
## Fixed Effects:
##
## Conditional model:
## (Intercept)
##       10.42
##
## Zero-inflation model:
## (Intercept)
##      -1.971
```

From which we can make a table of estimates as per Swihart, Fay & Miura (Journal of the American Statistical Association 2018).

```
##      Dataset      gamma             mu        pi     theta     alpha
## 1: Spz-Ctrls 10.4244783 33673.8981509 0.1393050 0.9364721 1.0678375
##       sig2_f    sig2_c
## 1: 0.2986621 0.0000002
```

The negative binomial model without zero-inflation and the zero-inflated Poisson model are nested within the ZINB model. The nesting involves parameter values on the boundary, so the proper likelihood ratio tests (LRTs) are non-standard. However, the standard LRT will be conservative, meaning that if they are

significant then the non-standard ones will be too. So, we straightforwardly conduct the standard LRTs:

```r
## negative binomial model without zero-inflation
spz.fit.xxnb <-glmmTMB(spz ~ 1+(1|feed)+(1|pint),
                       family="nbinom2",
                       ziformula=~0,
                       data=spz.supp ## controls only
)

##  zero-inflated Poisson model
spz.fit.zipo <-glmmTMB(spz ~ 1+(1|feed)+(1|pint),
                       family="poisson",
                       ziformula=~1,
                       data=spz.supp ## controls only
)


## standard LRTs
anova(spz.fit.zinb, spz.fit.xxnb)
```

```
## Data: spz.supp
## Models:
## spz.fit.xxnb: spz ~ 1 + (1 | feed) + (1 | pint), zi=~0, disp=~1
## spz.fit.zinb: spz ~ 1 + (1 | feed) + (1 | pint), zi=~1, disp=~1
##              Df    AIC    BIC  logLik deviance  Chisq Chi Df Pr(>Chisq)
## spz.fit.xxnb  4 4067.4 4080.4 -2029.7   4059.4
## spz.fit.zinb  5 3951.1 3967.3 -1970.5   3941.1 118.32      1  < 2.2e-16
##
## spz.fit.xxnb
## spz.fit.zinb ***
## ---
## Signif. codes:  0 '***' 0.001 '**' 0.01 '*' 0.05 '.' 0.1 ' ' 1
```

```r
anova(spz.fit.zinb, spz.fit.zipo)
```

```
## Data: spz.supp
## Models:
## spz.fit.zipo: spz ~ 1 + (1 | feed) + (1 | pint), zi=~1, disp=~1
## spz.fit.zinb: spz ~ 1 + (1 | feed) + (1 | pint), zi=~1, disp=~1
##              Df     AIC     BIC   logLik deviance   Chisq Chi Df
## spz.fit.zipo  4 4341770 4341783 -2170881  4341762
## spz.fit.zinb  5    3951    3967    -1971     3941 4337821      1
##              Pr(>Chisq)
## spz.fit.zipo
## spz.fit.zinb  < 2.2e-16 ***
## ---
## Signif. codes:  0 '***' 0.001 '**' 0.01 '*' 0.05 '.' 0.1 ' ' 1
```

Please consult the main manuscript and supplementary materials of Swihart, Fay & Miura (Journal of the American Statistical Association 2018) for more details on this models and LRTs.

To see how this fit on sporozoite controls would perform for sporozoite test data, the estimated line from the sporozoite control ZINB model for relating the mean of a COM to the standard deviation of a COM was plotted as the truth and the pair-wise data from the test data as points around that line and an R^2 was calculated as per Swihart, Fay & Miura (Journal of the American Statistical Association 2018). A value of 0.707 was obtained.
